# Facilitators and barriers to implementation of HPV vaccination in Tanzania: a mixed-methods study exploring perspectives from national, subnational, and community stakeholders, 2018–2023

**DOI:** 10.1016/j.vaccine.2025.127560

**Published:** 2025-08-01

**Authors:** Julie Garon Carlton, Doreen Pamba, Nessa Ryan, Willyhelmina Olomi, Nyanda Elias Ntinginya, Florian Tinuga, Lucas Maganga, Wiston William, Anange Lwilla, Emmanuel Kapesa, Joel Mwakisisile, Daniel Magesa, Andrea Mbunda, Jonathan M. Grund, Laura J. McCormick, Terri Hyde, Rebecca Casey

**Affiliations:** a U.S. Centers for Disease Control and Prevention, Global Health Center; Atlanta, GA, USA; b National Institute for Medical Research, Mbeya, Tanzania; c National Institute for Medical Research, Dar es Salaam, Tanzania; d Ministry of Health, Dodoma, Tanzania; e U.S. Centers for Disease Control and Prevention, Global Health Center; Dar es Salaam, Tanzania

**Keywords:** Human papillomavirus vaccine, Tanzania, LMIC, Program evaluation, Sustainability, Best practices

## Abstract

**Background:**

Cervical cancer is the fourth most common cancer among women globally, disproportionately affecting those in low- and middle-income countries (LMICs). In 2020, World Health Organization (WHO) Member States endorsed the 2030 Global Strategy toward Elimination of Cervical Cancer, recommending expanded access to human papillomavirus (HPV) vaccination. However, gaps remain in understanding how LMICs can sustain high HPV vaccine coverage. Tanzania, an early adopter among LMICs, introduced HPV vaccination into the national immunization schedule for 14-year-old girls in 2018 and achieved >90 % two-dose coverage by 2023. This study evaluated HPV vaccine program implementation in Tanzania, capturing stakeholder perspectives on barriers, facilitators, and recommendations.

**Methods:**

Stakeholders were interviewed in April 2024 in a concurrent mixed-methods evaluation. Participants included national and subnational immunization staff (*n* = 18), and health workers, teachers, and community influencers (*n* = 80). Four of 31 regions were purposively selected based on criteria including first-dose HPV coverage (2020–2022) and urban/rural distribution. Two health facilities were randomly selected from a list of facilities in each region, along with two schools administering the vaccine from each facility’s catchment area. Quantitative data were analyzed descriptively in STATA v.18, and qualitative data analyzed in ATLAS.ti Web (v19.3.1).

**Results:**

Political support, quality improvement cycles, and integration with existing systems were identified as contributing to program success. Funding gaps and staff shortages–particularly in regions with low HPV vaccination coverage–were among the reported barriers, along with poor coordination between health and education sectors and low community awareness. Recommendations included increasing government funding, strengthening cross-sector collaboration, training stakeholders, and expanding dissemination channels to improve demand and address vaccine hesitancy.

**Conclusions:**

Tanzania’s experience offers lessons for HPV vaccination in similar contexts. Addressing key barriers through increased funding, improved coordination, and enhanced community engagement could improve HPV vaccination implementation in Tanzania and elsewhere, contributing to global cervical cancer elimination.

## 1. Background

Cervical cancer was the fourth most common cancer globally among women in 2022, with more than half of the cases occurring in low and lower-middle income countries (LMICs) [1]. In 2020, the World Health Assembly adopted a global strategy to eliminate cervical cancer as a public health problem where all countries must reach 90 % of girls with human papillomavirus (HPV) vaccine by age 15 years by 2030 [2]. The number of countries with HPV vaccine in their national immunization schedules increased from four in 2006 to 133 in 2023, and with support of Gavi, the Vaccine Alliance, 16.3 million girls have been fully immunized since 2014 [3,4]. In 2022, based on data demonstrating efficacy of a single dose, the World Health Organization (WHO) recommended the option to use a one-dose HPV vaccination schedule instead of the previously recommended two-dose schedule for 9 to 20-year-old girls and young women, as well as young men [5]. This strategy has the potential to improve global HPV vaccination coverage dramatically. Gavi then invested in a revitalization of the program, supporting vaccine procurement, health system strengthening and technical support with plans to reach over 86 million girls with HPV vaccine by 2025 [6]. Gaps remain in understanding how countries, following national scale-up from HPV vaccine demonstration projects, can achieve and sustain high HPV vaccination coverage to reach the World Health Assembly’s targets.

In Tanzania, cervical cancer is the most common of all cancers among females, with 10,868 new cases and 6832 deaths estimated in 2022 [7]. Before introducing HPV vaccination nationally, Tanzania undertook a two-year demonstration project from 2014 to 2015 in seven districts of Kilimanjaro region, achieving over 93 % vaccination coverage for both doses (HPV1 and HPV2) in 9-year-old girls [8]. Tanzania was among the first Gavi-eligible countries to introduce HPV vaccine into the national immunization program, with Gavi eligibility based on gross national per capita income [9]. In April 2018, Tanzania officially launched a nationwide HPV vaccination program targeting girls aged 14 years. HPV vaccination is provided by the Ministry of Health (MoH) at health facility settings, community outreach posts, mobile clinics, and in-school settings. By 2019, the program had reached nearly 80 % first-dose coverage of 14-year-old girls through these strategies [10].

In 2019, a pilot project called *HPV-Plus* integrated HPV vaccination with other health initiatives, such as provision of deworming tablets, nutritional assessments, vision screening and educational components for both males and females aged 10–14 years in two districts, Makambako (in Njombe Region) and Ubungo (in Dar es Salaam Region). Notable increases in HPV vaccine uptake and completion rates were seen in the intervention districts, as well as a high level of acceptability of the program according to key stakeholders including adolescent girls, caregivers, program planners, and implementers [11]. Interventions such as *HPV-Plus* have demonstrated ways to complement other adolescent-centered services with vaccination programs, to increase coverage and completion of vaccination as well as improve access to these other essential services.

**Fig. 1.**
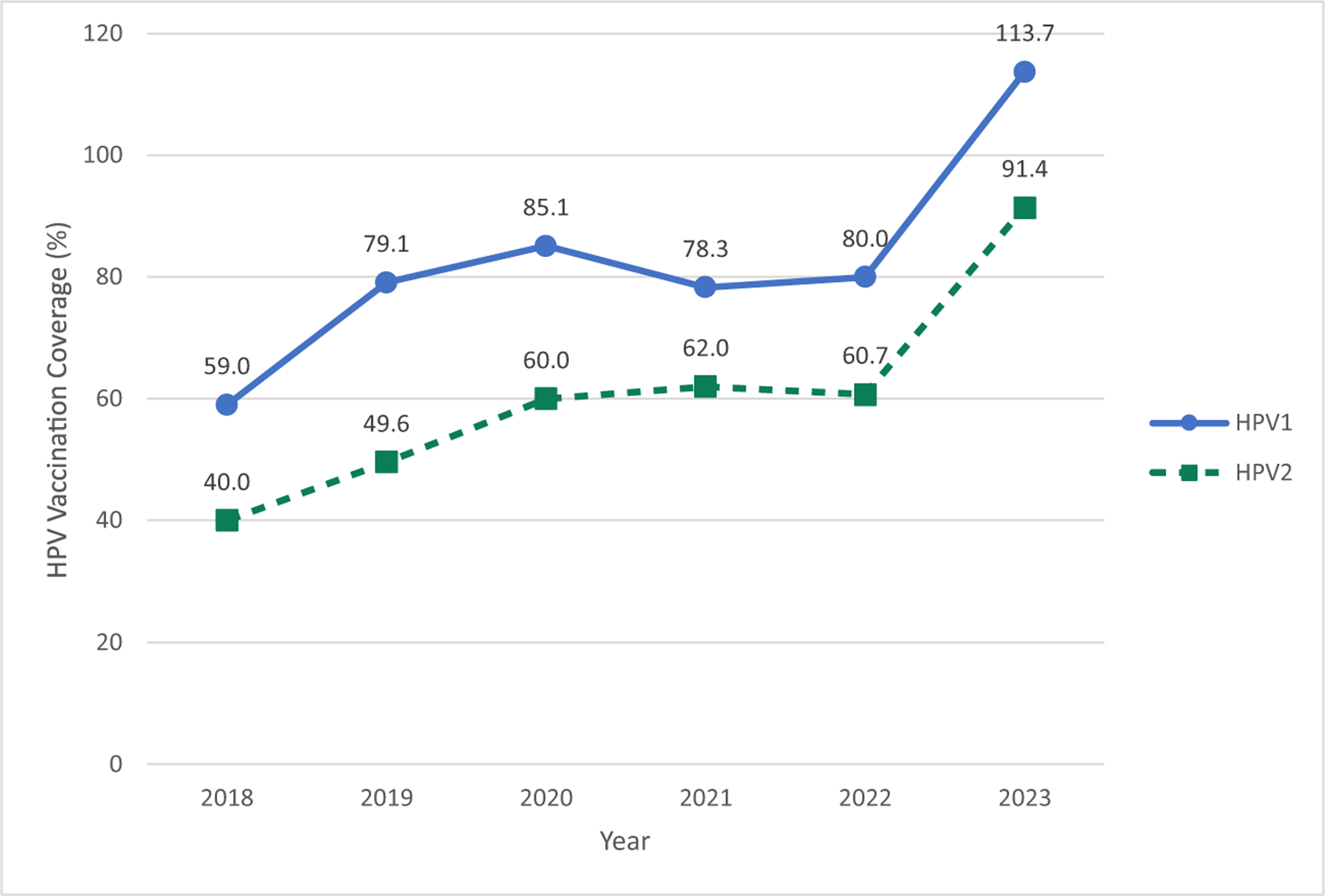
National HPV vaccination coverage among girls aged 14, 2018–2023, Tanzania.

**Fig. 2.**
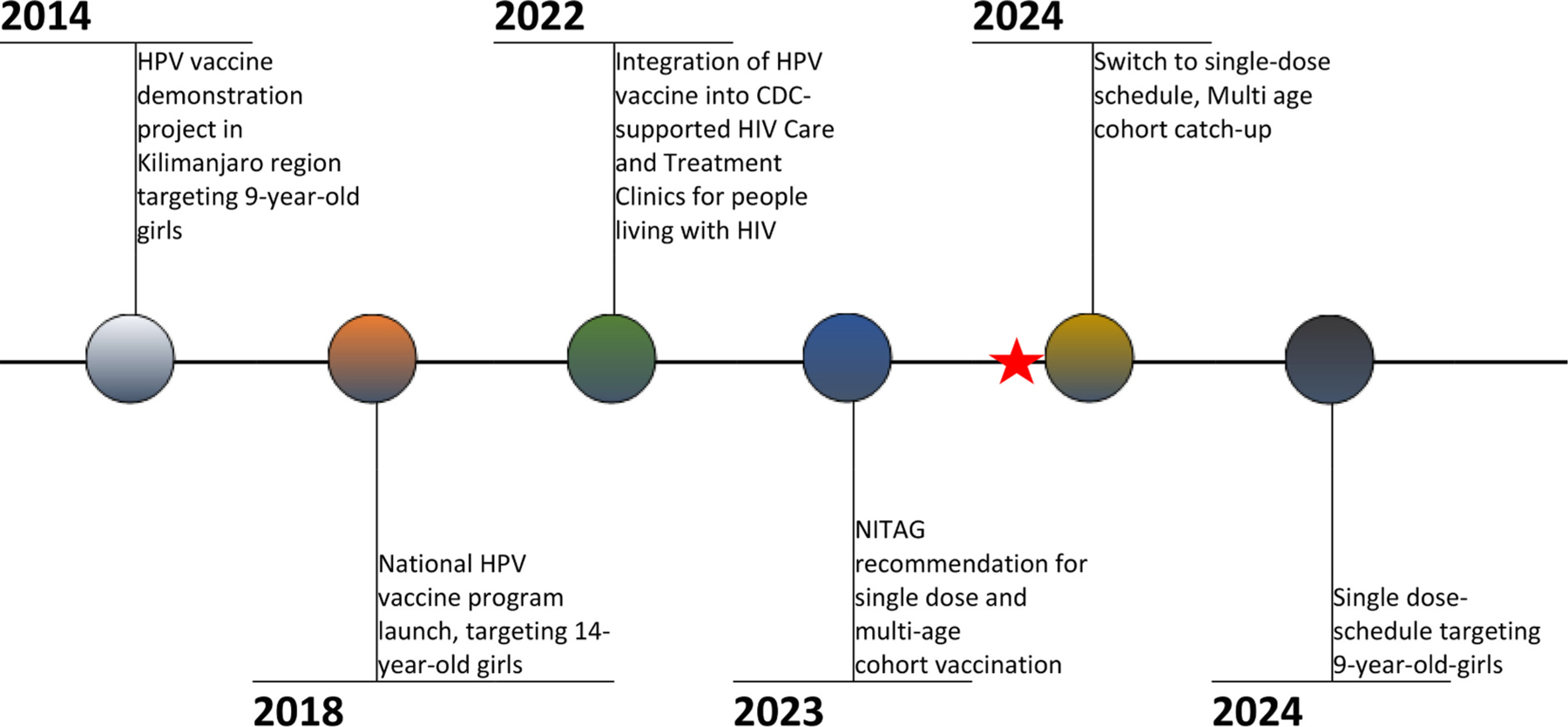
Timeline of HPV vaccine program in Tanzania. Evaluation data collection indicated by the star.

In 2023, HPV1 coverage derived from administration data was over 100 % nationally, likely due to underestimation of the target population or vaccination of girls older or younger than the target cohort of 14-year-old-girls (Fig. 1). HPV vaccination coverage increased overall from 2018 to 2023, with increases in later years likely due to intensification of overall vaccination efforts after the COVID-19 pandemic. However, poor follow up has resulted in some girls not completing the 2-dose series. In April 2024, the Tanzania MoH switched to a single-dose vaccination schedule, following the WHO recommendation, and conducted a multi-age cohort catch-up campaign. Girls living with HIV continue to receive 3-doses. Starting in 2025, Tanzania administers a single dose of HPV vaccine to girls aged 9-years-old through routine immunization that includes facility administration, and outreach modalities (school-based and community outreach). As most 9-year-old girls in Tanzania attend primary school, a transition to a younger target population allows the program to reach eligible girls more efficiently in fewer school-based vaccination sites. Follow up doses for girls living with HIV is incorporated into anti-retroviral treatment services to minimize stigma and missed doses. A timeline of key milestones in HPV vaccine implementation in Tanzania can be seen in Fig. 2.

Following the establishment of global cervical cancer elimination goals, there has been a dramatic increase in the number of countries implementing HPV vaccination programs. Many LMICs, including Tanzania, have rapidly scaled up national programs and achieved targets related to national vaccination coverage though geographic differences exist. In LMICs, knowledge is limited on evidence-based strategies to maintain high coverage rates over time; ensure equitable access across geographies, socioeconomic groups, and populations; and integrate immunization activities with other adolescent health services. We conducted a broad evaluation of Tanzania’s HPV vaccination program since introduction using methods that include literature and programmatic data review, key informant interviews, community interviews, survey of caregivers, and focus group discussions, with an aim of informing program improvement and integration efforts. Here we present findings from components of the larger evaluation, key informant interviews and community interviews, to describe the past barriers and facilitators to implementation, and future recommendations for sustainability of an established HPV vaccination program from the perspectives of national, subnational and community stakeholders in Tanzania.

## 2. Methods

### 2.1. Evaluation design and setting

This was a concurrent, mixed-methods study that included parallel data collection using secondary analysis of administrative coverage data derived from a desk review and primary data collection of exclusively qualitative key informant interviews and predominantly qualitative community interviews, with some close-ended questions. The quantitative results complimented the qualitative findings, with integration presented in the results section. The evaluation was conducted in four regions in Tanzania, namely Mbeya, Simiyu, Tabora, and Mtwara with primary data collection occurring between March–April 2024. These regions are in the Southern Highlands, Lake, Central, and Southern zones, respectively. Furthermore, HPV vaccines are routinely administered at Reproductive and Child Health clinics or HIV Care and Treatment Centers depending on HIV status of the beneficiary and the proximity between these locations within a facility.

### 2.2. Sampling and recruitment

For key informant interviews, a sample of six national and 12 subnational stakeholders (four regional-level and eight district-level) were targeted. At the national level, primary stakeholders, including representation from the MoH Immunization and Vaccine Development Program, were purposively selected to obtain background on the current HPV vaccination program and perceptions related to barriers and facilitators to implementation and implications around sustainability. Other individuals within the immunization program, Ministry of Education, and immunization partners from WHO, UNICEF, Clinton Health Access Initiative, and John Snow, Inc. were included to understand roles and responsibilities of key players within an established HPV program at the national level. Efforts were made to include a wide range of partners to best understand the breadth of contribution to the HPV vaccination program. National and subnational key informants were identified with the support of the MoH based on their involvement in HPV vaccination activities and included government, non-government stakeholders, implementing partners, and policymakers. At the regional level, four of Tanzania’s 31 administrative regions were purposively selected with MoH based on high (>80 %) and low (<60 %) administrative coverage of HPV1 from 2020 to 2023 and presence or absence of support from the U.S. Centers for Disease Prevention and Control or the U.S. President’s Emergency Plan for AIDS Relief. One urban and one rural district were selected within each of the four regions. Within each district, two health facilities were randomly selected from a list of all health facilities offering vaccination. One primary and one secondary school located within the health facilities’ catchment area were randomly selected from a complete list of all public primary and secondary schools within the district using a random number generator.

The target sample size for the community interviews was 16 health workers, 32 teachers, and 32 community influencers. Sampling strategy and target sample size were informed by WHO/UNICEF guidance on behavioral and social drivers of vaccination [12]. At sampled health facilities, the immunization health worker most closely involved in HPV vaccination was invited to participate in the interview. At sampled schools, the staff member most closely involved with HPV vaccination was invited to participate in the interview. Community influencers were identified with assistance from health facility staff and included local leaders (e.g. village executive officer, village chairperson, religious leaders, administrative representatives (e.g. councilor), traditional healers, community health volunteers, or other influential community members (e.g. lead mother in women’s group)).

### 2.3. Data collection

The desk review covered key areas of interest, including planning approaches, demand and communication platforms, behavioral and social drivers of vaccination, integration with other adolescent health interventions, delivery strategies, and considerations for sustainability. The documents included planning materials, program meeting reports, and implementation tools as well as relevant background in published and unpublished literature. HPV vaccination coverage data provided by the MoH were reviewed to describe trends in HPV vaccination over time. This information was used to inform the development of interview guides.

Key informant and community interview guides were pretested with stakeholders to ensure appropriateness, remove redundancies, and improve translation. The interview guides were further adapted, as needed, prior to data collection. Interviews were administered in Swahili, digitally recorded, transcribed verbatim and translated to English, with translations edited for clarity. Participants provided verbal consent before beginning the interview and the only personally identifiable information collected included job title and years in current role.

Key informant interviews among national and subnational stakeholders were conducted by two study staff, using a semi-structured interview guide in English or Kiswahili. One interview was conducted in-person and 17 by Zoom. Community interviews, conducted by trained interviewers at identified health facilities or schools, assessed health worker, teacher, and community leader behaviors, attitudes, and perceptions around HPV vaccination. Community interview guides included closed-ended questions on HPV and HPV vaccine knowledge, HPV vaccine acceptability and attitudes on recommending HPV vaccine, as well as open-ended questions on overall best practices and challenges from the perspective of the stakeholder. Community interviews were administered by trained interviewers with closed-ended responses recorded using android based devices equipped with Open Data Kit [13] software.

### 2.4. Analytical framework

**Fig. 3.**
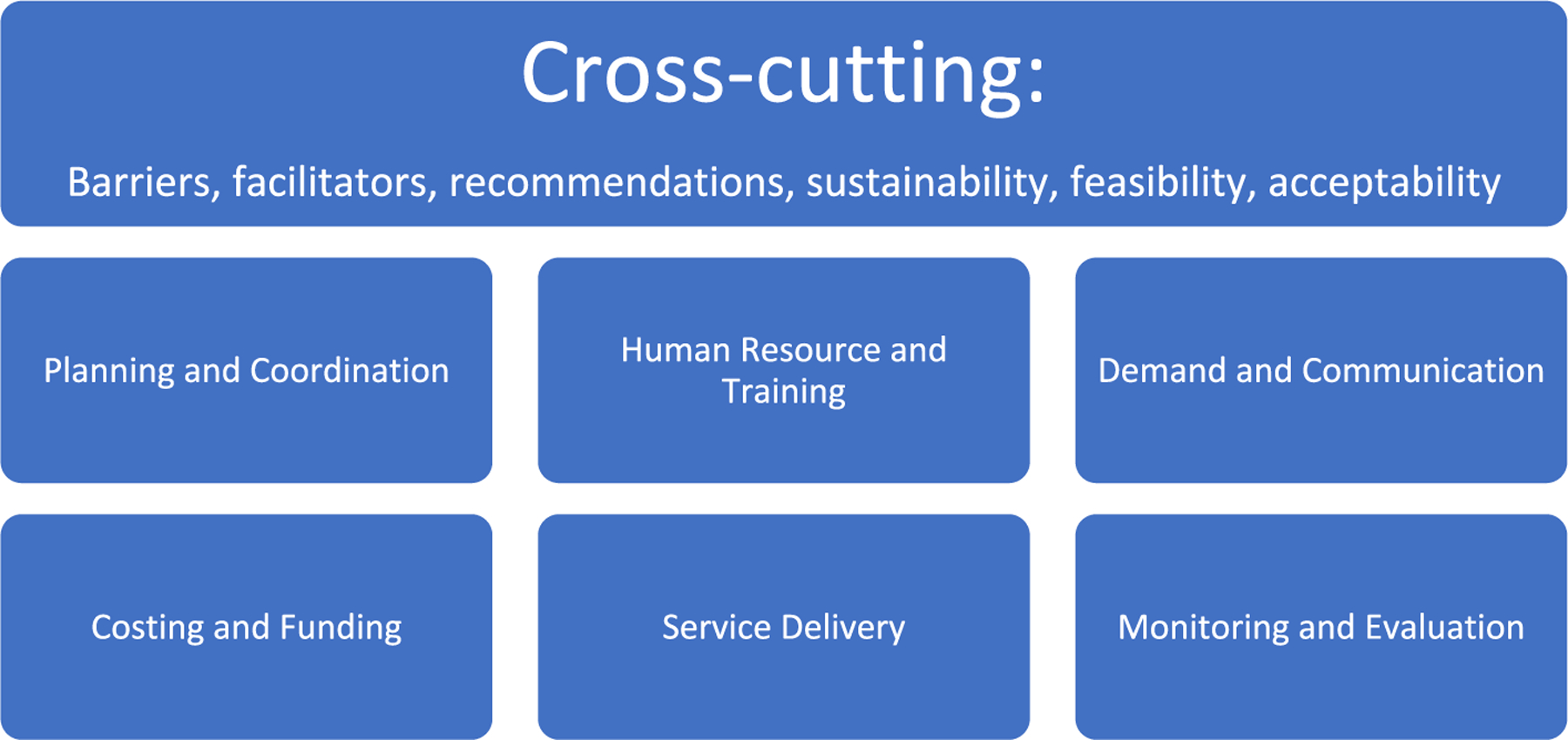
Analytical Framework developed for evaluation of HPV vaccine implementation in Tanzania.

The Framework Method was used to guide analysis of qualitative data [14], which is an approach to thematic analysis of qualitative data. After initial identification of categories based on main research questions, researchers developed a working analytical framework, informed by existing immunization frameworks such as Behavioral and Social Drivers of Vaccination and WHO Guide for Conducting an Expanded Program on Immunization Review [12,15], that was then applied to subsequent transcripts using existing codes. Data were charted into a framework matrix and then interpreted. The framework used in this analysis can be seen in Fig. 3.

### 2.5 Data synthesis and analysis

Transcripts were analyzed through a team-based thematic analysis approach consisting of deductive (extracted from research and interview guide questions) and then inductive coding (developed through initial review of transcripts). An initial set of codes and framework were developed from the interview guides. Two transcripts of each stakeholder type were purposively selected for thematic analysis and codes were adapted to incorporate additional topics and themes. After piloting and revising the codebook on a sample of transcripts, ATLAS.ti Web (Version 19.3.2) [16] was used by the study team to code all transcripts within the established framework.

Frequency tabulations were made for data collected from closed-ended questions from interviews with health workers, teachers and community influencers, focused on perceptions and experience with the HPV vaccination program. Descriptive statistics (e.g., totals, proportions) were calculated for key close-ended questions for each stakeholder type and for the overall sample in the community interviews. Quantitative data was analyzed using STATA v.18 [17].

## 3. Results

### 3.1. Participant characteristics

A total of 98 participants were included in the evaluation via 18 key informant interviews (*n* = 6 national, *n* = 12 subnational) and 80 community interviews (*n* = 17 health workers, *n* = 31 teachers, and *n* = 32 community influencers). National stakeholders were interviewed in Dodoma and Dar es Salaam (n = 6), while the remaining participants (*n* = 92) were in the study regions, Mbeya, Mtwara, Simiyu, and Tabora (Table 1). In evaluating the implementation of the HPV vaccination program in Tanzania, responses were grouped into several categories, including barriers to program implementation, facilitators to program implementation, and recommendations to improve the HPV vaccination program. Table 2 provides a summary of stakeholder perspectives from key informant interviews with national/subnational staff and the qualitative component of the community interviews, by category.

### 3.2. HPV vaccination program implementation barriers

Participants were asked about challenges to achieving and sustaining at least 90 % coverage with one dose of HPV vaccine for all eligible girls in Tanzania. According to national and subnational key informants, issues related to funding were mentioned as top barriers to HPV vaccine implementation. This included mentions, among key informant interviews from low performing regions, of the overall amount of funding leading to gaps in funds for transportation or incentives for staff, timeliness in receipt of funding, and dissemination to the subnational level. National and subnational stakeholders from all areas mentioned related concerns about long-term sustainability of the program. As one national stakeholder mentioned about funding challenges, *“This program is supported by* Gavi*, so how [can the] country stretch itself to ensure that it [can] support? That is a challenge if it happens that Gavi says ‘We don’t give [in the future].’ Are we ready to support the program? To me, I think that is [a] challenge.”*

Other barriers to achieving high coverage included poor coordination between health workers and teachers to deliver services, e.g. limited sharing of information on planned vaccination dates, preparation timelines or data sharing expectations. Staffing issues such as health worker shortages or high turnover among program implementers was also raised as a challenge. Without additional staff recruited specifically for HPV-related activities, health facility staff were expected to visit schools for HPV vaccination activities while maintaining routine services at facilities. As one health worker from a region with low coverage stated during the community interview, “*The first challenge we are experiencing is having a few number of providers, so we have many responsibilities and sometimes we fail to deliver on time. We are only two…so sometimes we have a very limited time to deal with HPV vaccine.*”

Lastly, lack of awareness and low demand for HPV vaccine in the community was mentioned as a challenge. Examples of this include limited knowledge in communities about cervical cancer and the benefits of HPV vaccine and a lack of engagement of parents in the vaccination process. When interviewed, one teacher mentioned, “*The challenge is when they do not have information about this vaccine. They are only told by their children that tomorrow there’s HPV vaccination but themselves do not know about the vaccines…”.* Specifically, hesitancy among caregivers and rumors of infertility were cited as contributors to low HPV vaccine uptake. Another teacher said, *“these parents rely on unfounded beliefs like, these vaccines they’re bringing might reduce fertility or might harm our children in the future”.* This was supported in the quantitative data as most health workers (62 %), teachers (84 %), and community influencers (84 %) reported hearing rumors about the HPV vaccine. The rumors included that the HPV vaccine may affect a girl’s fertility, cause cervical cancer, or is not safe (Table 3).

### 3.3. HPV vaccination program implementation facilitators

Participants were asked what makes the provision of HPV vaccine a success since the program was launched in 2018 and which strategies have been most successful for reaching target populations. Several themes related to best practices and facilitators to success emerged during key informant interviews. Informants mentioned political will and committed immunization staff being crucial to the success of the HPV vaccination program. One national-level stakeholder said, *“…A thing that has helped us since the beginning is the political support, unlike other vaccines apart from the COVID-19 vaccine, the HPV vaccine was launched by … the Vice President of the United Republic of Tanzania* [Samia Suluhu Hassan]*. So, the act of a woman launching this, and a woman who later became the President, created significant political awareness about the HPV vaccine*“.

At an operational level, quality improvement cycles, or ongoing monitoring and regular review of lessons learned from high and low performing subnational areas, helped inform program improvement efforts. For example, participants mentioned lessons learned on reasons for vaccine hesitancy among key populations in low performing areas helped inform messaging and communication strategies as the program evolved. Additionally, national-level stakeholders claimed integrating the HPV program with existing implementation platforms (e.g., school health platforms, HIV Care and Treatment Clinics) and program activities (e.g., National Health Days) helped leverage established health systems to initiate new immunization activities. Key informants and interview participants at all levels emphasized the importance of engaging with community leaders to identify out-of-school girls and using multiple, evidence-based sources of information to create awareness around HPV vaccination and program activities.

Reported facilitators to achieving high HPV program success included strong coordination within the health system and partners (e.g. subnational immunization officers, local NGOs involved in immunization), engaging community leaders (e.g., religious leaders, village chiefs), creating increased awareness of the HPV vaccination program among the community, and using trusted sources of information (e.g. health workers, religious institutions) to disseminate messages about the program (Table 2). Providing education to both parents and girls was mentioned by all interviewees as a way to overcome challenges related to hesitancy and low demand for vaccination. One teacher said, *“I am a believer in education, I believe that to have a community that listens and accepts something, it must receive sufficient education”.*

Quantitative data collected during community interviews supported findings related to program implementation. Nearly all community stakeholders felt that HPV vaccination is important (97 % of health workers, 94 % of teachers, and 100 % of community influencers) and felt confident recommending HPV vaccine to girls and caregivers (97–100 % of health workers, teachers, and community influencers). Teachers (63 %), community leaders (56 %), and health workers (47 %) were most frequently listed as the best channels of communication to reach caregivers and girls followed by radio (39 %), TV (23 %), and written communication sources (17 %) (Table 3).

### 3.4. Recommendations to improve HPV vaccine coverage and sustain success

All participants were asked what they would do to improve HPV vaccination services in Tanzania, if they had the chance. Key recommendations from national-level key informants included increasing government funding to support the HPV vaccination program to ensure sustainability in the event donor support is withdrawn in the future. National-level stakeholders also recommended improving collaboration between health and education sectors at all levels. As schools are a key component of the HPV vaccination strategy, ensuring teachers and school administrators were informed, involved in the planning and implementation process, and actively engaged in HPV related communications and activities was seen as an important recommendation by immunization staff. One national stakeholder mentioned, *“We have to make sure there is good cooperation between health and education sector[s], so that we can get our targeted candidates in schools. We have to strengthen planning and implementation between these two areas.”*

Ongoing refresher courses and increased supervision for healthcare workers, teachers, and community health workers, was recommended by both subnational interviewees and community interview respondents. Regular seminars were suggested to reinforce learning for program implementers and address employee turnover. Since teachers represent an integral interface with girls, providing specialized training to teachers specifically will in turn, ensure accurate information is being relayed to girls and caregivers. A sub-national level stakeholder from a region with high HPV coverage articulated, *“We need to train health teachers too because they are also helping us and if a teacher has negative attitude toward a vaccine, it’s very hard to reach girls in school because not all teachers understand the importance of [the] vaccine.”*

**Fig. 4.**
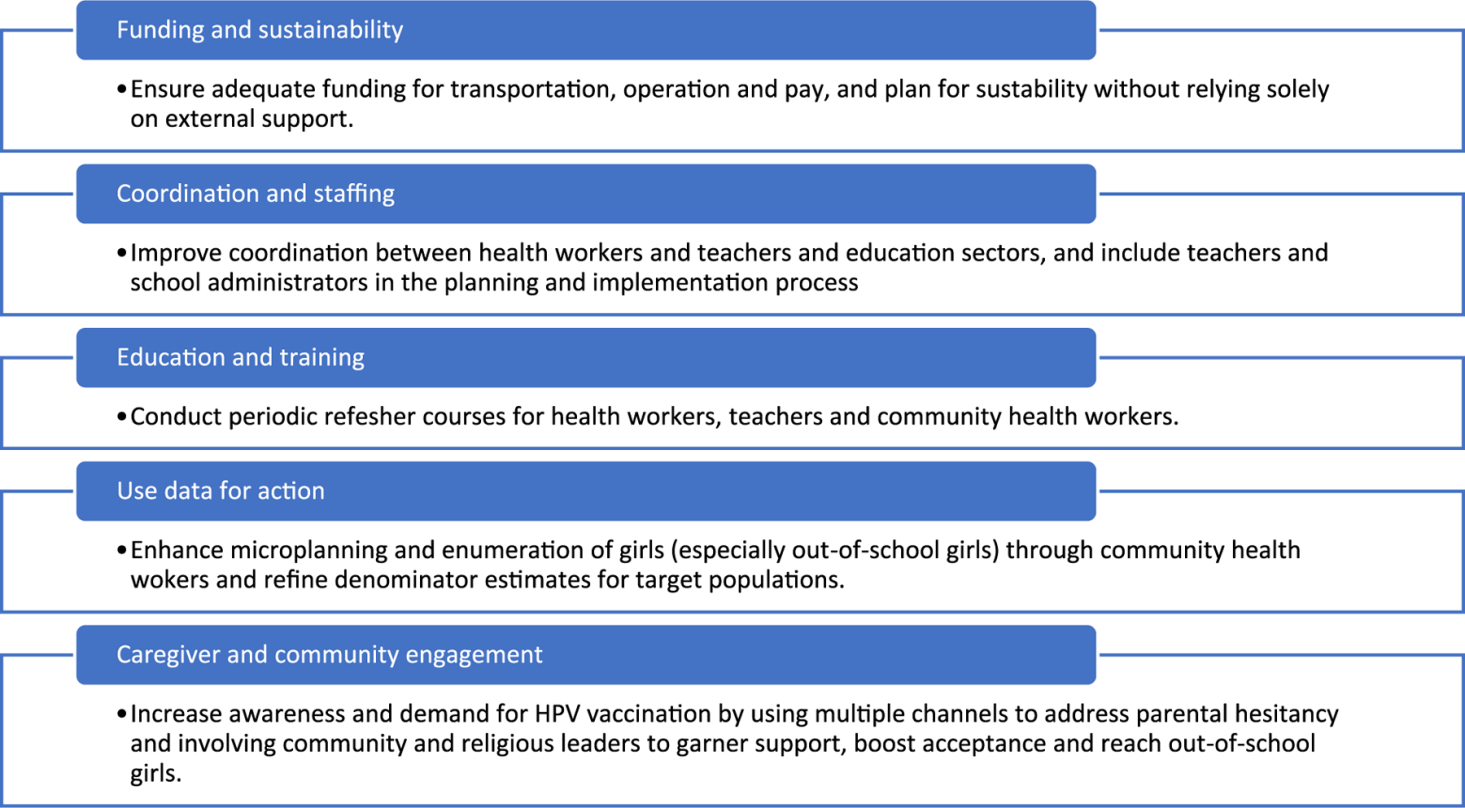
Key lessons learned for successful implementation of a national HPV vaccination program.

Additionally, subnational stakeholders recommended advocating for using data for action in ways such as improving the estimated number of girls in the priority population in the region used as a program target to guide program planning. Improved microplanning and better enumeration of girls (denominator estimates), including utilizing community health workers to identify out-of-school girls, was mentioned to improve program implementation. In addition, community stakeholders recommended, to pre-empt hesitancy that may arise with a new program, a focus on reaching caregivers with information about the HPV vaccination program and on improving engagement with the community, particularly community and religious leaders (e. g., meetings with local government leaders, public sessions in villages, sensitize women visiting health clinics). Lastly, expanding information dissemination channels (e.g., media, radio, billboards, community meetings) will also ensure that messages reach key beneficiaries. Fig. 4 summarizes overall stakeholder identified key lessons learned for successful implementation of the national HPV vaccination program in Tanzania.

## 4. Discussion

Five years after national introduction, stakeholders identified political will and partnerships as key to the successful implementation of the HPV vaccination program over time, referencing lessons learned from earlier phases of the program or from information gathering in specific geographic areas. With strong coordination within the health sector and efforts to integrate HPV vaccine into existing health platforms and activities, such as HIV programming, Tanzania’s HPV vaccination program was able to build on strengths of the routine immunization program, a strategy that may further generate social and economic benefits [18]. Our evaluation highlights the sustained operational success of the program, continuing to reach the target population with lifesaving vaccines by creating strong community awareness of the program, educating caregivers and adolescents, and engaging with community leaders.

Challenges remain, including combating vaccine hesitancy, particularly as it relates to infertility concerns, and low demand for HPV vaccination in the community. Studies in other sub-Saharan African countries have confirmed that the sexual and reproductive context of HPV and cervical cancer can enable misinformation related to HPV vaccine [19–21]. Exposure to rumors, fear of adverse effects, and lack of awareness are documented sources of hesitancy to HPV vaccine and poor uptake in LMICs [22,23]. Funding challenges, competing priorities and limited staff for carrying out HPV-vaccine related activities were also identified as challenges. Integrating vaccination services with other desired adolescent health services, and the adoption of single-dose schedules, thus reducing costs and the need for follow up, may begin to mitigate some of these inherent difficulties. [24,25] With increased access to available vaccines against vaccine-preventable diseases, there has been a considerable escalation in the number of vaccines introduced into national immunization schedules in LMICs, straining the already overworked health workforce [26,27]. Increased government funding for staffing needs, improved training and capacity building, and expanded use of community health workers may help alleviate some health workforce strains [28,29].

Stakeholders in this evaluation recommended ongoing health worker and teacher training, integration of the HPV vaccination program with other health services, expansion of dissemination channels to reach beneficiaries with accurate information about HPV vaccines, and improved engagement with the community. A qualitative study evaluating an enhanced and integrated, school-based HPV vaccination program in two districts in Tanzania found stakeholder knowledge levels around HPV, cervical cancer, and HPV vaccines were a key factor in HPV vaccine acceptability [11]. As knowledge gaps are known to exacerbate barriers to uptake of HPV vaccine, reaching multiple types of stakeholders with consistent, tested, and targeted messaging may help overcome these barriers [30–32].

This cross-sectional evaluation captured perspectives and attitudes at the time of the interview, thus changes in opinion could not be captured over time. Additionally, some questions in the interview guides referred to events early in Tanzania’s experience with HPV vaccination, likely contributing to recall bias in the findings. Additionally, there is the possibility of social desirability bias if participants provided responses that might be seen as favorable to interviewers. To mitigate this risk, data collectors were trained to build rapport with participants before starting the interview and provide information to participants that responses provided were confidential. Administrative coverage data was limited in granularity, making inferences about subgroups or subnational differences not possible. Lastly, while the perspective of the main beneficiary of the HPV vaccination program, girls, was included in the larger evaluation project, findings are not reported in this paper. Despite this context and these limitations, the inclusion of a diverse sample including different key informants related to the HPV vaccination program, high- and low-performing regions in the purposeful selection of the sample, and triangulation of different data types enabled this evaluation to draw conclusions related to overall barriers, facilitators, and recommendations in Tanzania.

## 5. Conclusions

Achieving sustained, high HPV vaccination coverage requires adequate funding, strong coordination between health and education sectors, use of high-quality data to inform implementation, and ongoing training support for health workers and teachers. Multiple messaging channels and effective utilization of caregivers, community and religious leaders can improve awareness and increase demand for HPV vaccination among beneficiaries. In addition to informing Tanzania’s immunization program, findings from this evaluation may contribute to efforts toward sustaining HPV vaccination coverage in other settings facing similar challenges. Lessons learned from experiences in early adopting countries can inform policy-making decisions and guide implementation strategies at a broader scale. Tanzania’s successful HPV vaccination program is a crucial step toward achieving cervical cancer prevention and elimination goals at the national and global levels.

## Figures and Tables

**Table 1:** Stakeholders by group (*n* = 98).

Study Component	Stakeholder Group	National	Region				Total
			Mbeya	Mtwara	Simiyu	Tabora	
Key Informant Interviews	National/subnational key informants	6	3	3	3	3	18
Community Interviews	Health Workers	–	4	5	4	4	17
	Teachers	–	8	7	8	8	31
	Community Influencers	–	8	8	8	8	32
	Total	6	23	23	23	23	98

**Table 2:** Stakeholder perspectives on program barriers, facilitators and recommendations.

Category	Key themes from interviews with national and subnational immunization staff	Quotations from community interviews
Barriers to program implementation	• Funding related issues (lack of funds for transportation, incentives, sustainability without Gavi support) • Poor coordination between health workers and teachers to deliver services, health worker shortages/turnover • Lack of awareness and low demand for HPV vaccine • Hesitancy among parents and infertility rumors	*Hesitancy around HPV vaccination* “The challenge comes from parents’ understanding. Some parents have misconceptions; when you tell them that children need to be vaccinated, they start thinking about different things, like these vaccines might harm their children in the future. That’s where the difficulty arises.” - Teacher *Lack of awareness in the community* “Adults don’t go to school, so they may not know if the children won’t inform them, that’s why I see the information about HPV wasn’t much disclosed to people, only few people are aware about it” - Community Leader *Low demand for immunization* “First, no parent really follows up to see if their child got the vaccine or not, and also for them as parents, it’s not... I mean, I could say that it’s not really a very important thing for them. So whether the child gets the vaccine or not, as a parent, they don’t really bother.” - Teacher *Competing priorities and limited staff* “The first challenge we are experiencing is having a few number of providers, so we have many responsibilities and sometimes we fail to deliver on time, we are only two. So sometimes we have a very limited time to deal with HPV vaccine” - Health Worker
Facilitators to program implementation	• Political will and committed health staff • Learning from experience - sharing lessons from high and low performing areas • Integrating with existing health systems/platforms/activities • Engaging with community leaders to identify out-of-school girls • Using multiple information channels to create awareness	*Strong community awareness of HPV program* “Education, at first, they were not aware so they had so many questions but after we have talk to them and reassure them that this vaccine is safe, they don’t worry anymore” - Health worker *Good coordination within the health system and schools* “. if you want to have smooth exercise health teacher and health care providers should work as one team” - Health worker *Efforts to educate parents* “For those challenges [with reaching girls], I think its only to educate parents, if we educate them on the importance of HPV vaccine, they will understand and cooperate, if we give them real examples, they will positively respond just like other vaccines.”- Teacher *Engaging with community leaders* “you need also to educate community leaders such as ward executive officers, street chairpersons, if you uses community leaders, its very easy to reach girls” - Health worker *Using effective sources of information on HPV* “Something that I think might be helpful is to advertise from the churches, at mosques and other public places.” - Community Influencer
Recommendations to improve the HPV vaccination program	• Increase government funding to support HPV vaccination program • Implement refresher courses for health workers, teachers, and community health workers; increased supervision • Improved collaboration between health and education sectors (both at national ministry level and individual health facility/school level) • Improved denominator estimates, better enumeration of girls, and utilization of community health workers • Engage media and religious leaders to increase community awareness	*Focus on reaching caregivers with information* “The main thing, I think, is to advise, to hold meetings with parents, and especially provide education to parents, because even the health workers have their role, but the biggest challenge they face comes from the parents. So the key thing is to provide education to parents.” -Teacher *Improve engagement with the community* “The first thing, I would communicate with village leaders so that every leader would educate his village since they are close with their people.” - Health Worker *Expand information dissemination channels* “...provide education through radio, television and also in community meetings, to use billboards to educate community on the importance of hpv vaccine” - Health worker *Maintain ongoing training for health workers and educate teachers* “we should have regular seminars to experts due to changes on the doses, and we know every year new providers are being employed and you will find new employees are being given the facility while she doesn’t know anything about HPV vaccine” - Health worker “Personally, I would like education to be provided to health teachers at schools, since we are the ones responsible at registering children, informing them and make sure they are available on the vaccine’s day, so our health education isn’t that sufficient since we didn’t specialize in health issues” - Teacher

**Table 3:** Perspectives on HPV vaccine according to stakeholders participating in the community interviews, Tanzania - 2024.

	Health worker (N = 32)	Teacher (*N* = 32)	Community influencer (N = 32)	Total (*N* = 96)
	n (%)	n (%)	n (%)	n (%)
Thinks HPV vaccine is very important for girls to receive	31 (96.9)	30 (94)	32 (100)	93 (97)
Feels confident recommending HPV vaccine to girls and caregivers	32 (100)	31 (97)	32 (100)	95 (99)
Reported having heard rumors about HPV vaccine	20 (63)	27 (84)	27 (84)	74 (77)
Rumors heard*				
*HPV vaccine will affect girl’s fertility*	18 (90)	22 (81)	24 (89)	64 (67)
*HPV vaccine will cause cervical cancer*	4 (20)	5 (19)	3 (11)	12 (13)
*HPV vaccine will cause severe side effects*	1 (5)	5 (19)	6 (22)	12 (13)
*HPV vaccine is not safe*	2 (10)	7 (26)	2 (7)	11 (11)
*HPV vaccine is experimental*	2 (10)	0 (0)	0 (0)	2 (2)
Best communication channel for reaching girls about HPV vaccination*				(*N* = 64)
*Teachers*	23 (72)	17 (53)	–	40 (63)
*Community leaders* e.g.*, local community authorities*	14 (44)	22 (69)	–	36 (56)
*Health worker*	17 (53)	13 (40)	–	30 (47)
*Radio*	10 (31)	15 (17)	–	25 (39)
*Community health workers* e. g.*, CHBCs*	10 (31)	10 (31)	–	20 (31)
*TV*	6 (19)	9 (28)	–	15 (23)
*Pamphlets/leaflets*	2 (6)	9 (28)	–	11 (17)
*WhatsApp/Social media*	0 (0)	5 (16)	–	5 (8)
*Religious leaders*	1 (3)	0 (0)	–	1 (2)

* Respondents could select multiple choices.

## Data Availability

Data will be made available on request.
